# Genetic Code Expansion for Controlled Surfactin Production in a High Cell-Density *Bacillus subtilis* Strain

**DOI:** 10.3390/microorganisms13020353

**Published:** 2025-02-06

**Authors:** Alexander Hermann, Eric Hiller, Philipp Hubel, Lennart Biermann, Elvio Henrique Benatto Perino, Oscar Paul Kuipers, Rudolf Hausmann, Lars Lilge

**Affiliations:** 1 Department of Bioprocess Engineering, Institute of Food Science and Biotechnology, University of Hohenheim, 70599 Stuttgart, Germany; alexander.hermann@uni-hohenheim.de (A.H.); eric.hiller@uni-hohenheim.de (E.H.); lennart.biermann@uni-hohenheim.de (L.B.); eperino@uni-hohenheim.de (E.H.B.P.); rudolf.hausmann@uni-hohenheim.de (R.H.); 2 Core Facility Hohenheim, Mass Spectrometry Core Facility, University of Hohenheim, 70599 Stuttgart, Germany; philipp.hubel@uni-hohenheim.de; 3 Department of Molecular Genetics, University of Groningen, 9747 AG Groningen, The Netherlands; o.p.kuipers@rug.nl

**Keywords:** *Bacillus subtilis*, surfactin, biosurfactants, genetic code expansion, non-canonical amino acid, bioprocess engineering, bioreactor, molecular adaptation, proteomics

## Abstract

Background: In biotechnology, *B. subtilis* is established for heterologous protein production. In addition, the species provides a variety of bioactive metabolites, including the non-ribosomally produced surfactin lipopeptide. However, to control the formation of the target product-forming enzyme, different expression systems could be introduced, including the principle of genetic code expansion by the incorporation of externally supplied non-canonical amino acids. Methods: Integration of an amber stop codon into the *srfA* operon and additional chromosomal integration of an aminoacyl-tRNA synthetase/tRNA mutant pair from *Methanococcus jannaschii* enabled site-directed incorporation of the non-canonical amino acid O-methyl-L-tyrosine (OMeY). In different fed-batch bioreactor approaches, OMeY-associated surfactin production was quantified by high-performance thin-layer chromatography (HPTLC). Physiological adaptations of the *B. subtilis* production strain were analyzed by mass spectrometric proteomics. Results: Using a surfactin-forming *B. subtilis* production strain, which enables high cell density fermentation processes, the principle of genetic code expansion was introduced. Accordingly, the biosynthesis of the surfactin-forming non-ribosomal peptide synthetase (NRPS) was linked to the addition of the non-canonical amino acid OMeY. In OMeY-associated fed-batch bioreactor fermentation processes, a maximum surfactin titre of 10.8 g/L was achieved. In addition, the effect of surfactin induction was investigated by mass spectrometric proteome analyses. Among other things, adaptations in the *B. subtilis* motility towards a more sessile state and increased abundances of surfactin precursor-producing enzymes were detected. Conclusions: The principle of genetic code expansion enabled a precise control of the surfactin bioproduction as a representative of bioactive secondary metabolites in *B. subtilis*. This allowed the establishment of inducer-associated regulation at the post-transcriptional level with simultaneous use of the native promoter system. In this way, inductor-dependent control of the production of the target metabolite-forming enzyme could be achieved.

## 1. Introduction

In microbial biotechnology, *Bacillus subtilis* has been established as a platform organism for the bioproduction of various target products [1]. As an example, *B. subtilis* is used as a super-secreting cell factory for the production of customized enzymes [2]. In addition, the *B. subtilis* family exhibits a large number of gene clusters that encode the biosynthesis of bioactive secondary metabolites with antimicrobial properties [3,4]. Accordingly, *B. subtilis* has already been applied as a host for the recombinant production of bioactive metabolites [5].

Surfactin serves here as a well-known lipopeptide that is natively produced by several *Bacillus* strains. Due to its structural properties, which consist of a cyclic peptide moiety (L-Glu, L-Leu, D-Leu, L-Val, L-Asp, D-Leu, L-Leu) attached to a β-hydroxy fatty acid chain of varying length [6], surfactin exhibits bioactivities, including antimicrobial and anticancer activities [7,8], but also emulsifying and foaming activities, making surfactin a powerful microbial biosurfactant [9]. In this context, an increasing number of patents and articles with the terms ‘cosmetic’ and ‘surfactant’ were found in the Espacenet database, which emphasize the potential of the biosurfactant surfactin in cosmetic applications [10]. Furthermore, the combination of high safety, molecular stability and antimicrobial activity makes surfactin a promising substance for both food preservation and agricultural applications [11,12]. As the structural properties of surfactin make it a tensioactive biomolecule, applications have also been found in microbial-assisted oil extraction [13].

The production of surfactin is based on a multi-modular enzyme complex, the non-ribosomal peptide synthetase (NRPS) SrfA, which is encoded by the *srfAA-AD* operon. Due to the metabolic resources required for the NRPS expression and the subsequent surfactin biosynthesis, the surfactin production shows a detectable impact on cell growth [14,15]. Accordingly, the NRPS biosynthesis is controlled by different regulatory networks, including various key cell differentiation modulators [16,17,18,19]. At the posttranslational level, the surfactin-forming NRPS is activated by the 4-phosphopantetheinyl transferase Sfp before surfactin is intracellularly produced and secreted [20,21].

In adapted high cell-density bioreactor processes using the non-sporulating *B. subtilis* strain BMV9, a surfactin titer of 36 g/L could be achieved [22]. However, although *B. subtilis* is able to grow in the presence of surfactin of up to 100 g/L, the associated antimicrobial properties also show negative feedback effects on the production strain [11,23]. Accordingly, metabolic strain engineering and adapted bioprocess design were required to establish a suitable production pipeline for surfactin [24]. In particular, the surfactin production could be improved by systematical strain engineering targeting metabolic precursor pathways, molecular regulatory networks and tolerance mechanisms [18,19,25]. In addition, adapted bioprocesses were established for a more efficient bioreactor-based surfactin production. For example, oxygen-dependent decoupling of biomass formation and surfactin production was demonstrated [15], while an external foam column was established for in situ product removal in aerated surfactin production processes [26]. Moreover, high cell-density fermentation processes were implemented by using sporulation-deficient *B. subtilis* strains and adjusting the feeding-rate, which has a significant impact on the microbial productivity [22,27].

Another strategy for improving the surfactin production in *B. subtilis* strains is the modification of the expression level of the native *srfA* promoter. In this context, the influence of the ComX-dependent *B. subtilis* quorum sensing system, a key regulatory pathway affecting surfactin production, was characterized and modeled [28,29]. However, substitution of the native *srfA* promoter by the endogenous constitutively active P*_veg_* promoter did not lead to an increase in surfactin concentration in all tested *B. subtilis* strains, indicating differences in regulatory fine-tuning between weak and strong surfactin production strains [30].

However, orthogonal protein translation based on the expansion of the genetic code offers another option to control the bioproduction of target substances. For this purpose, orthogonal aminoacyl-tRNA synthetase (aaRS)/tRNA pairs have to be integrated into the expression host, leading to site-directed incorporation of non-canonical amino acids (ncAA) into the target proteins [31,32]. In this context, the amber stop codon (UAG) is commonly used for orthogonal tRNA design as it is the least abundant codon sequence in bacteria, reducing the non-specific effects on the proteome of the expression host [32]. Accordingly, codon substitutions are used to introduce the amber stop codon in order to generate a tunable protein translation system with low leakage, as previously shown for the microbial expression hosts *E. coli* and *B. subtilis* [32,33,34]. Exemplarily, an orthogonal translation system based on genetic code expansion was established in *B. subtilis*, in which an aaRS/tRNA mutant pair derived from *Methanococcus jannaschii* was used for incorporation of the ncAA O-methyl-L-tyrosine (OMeY). In this way, an OMeY-mediated balance of cell growth and N-acetylneuraminic acid was achieved [32].

**Figure 1:**
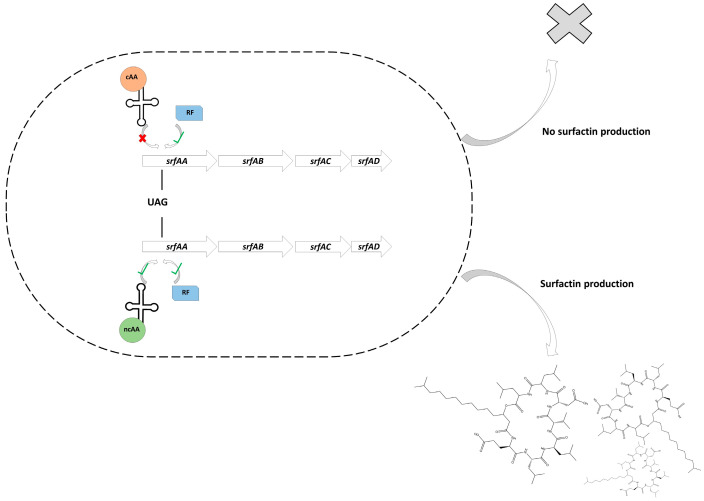
The principle of surfactin biosynthesis based on genetic code expansion. Under control conditions, a canonical amino acid (cAA) is incorporated into the nascent polypeptide chain, while the release factor (RF) recognizes the stop codons, such as the amber stop codon (UAG), which leads to an end of the translation process. By introducing an orthogonal aaRS/tRNA system, both the release factor and the incorporation of a non-canonical amino acid (ncAA) are able to target the amber stop codon.

In this study, the principle of orthogonal protein translation based on genetic code expansion was applied to the ncAA-based production of surfactin, a representative of the bioactive secondary metabolites with antimicrobial character produced by *B. subtilis* (Figure 1). Therefore, the *srfAA* gene within the *srfA* operon was selected for the integration of an amber stop codon, while the chromosomal integration of an aaRS/tRNA mutant pair allowed the incorporation of OMeY. In this way, OMeY-dependent NRPS translation enabled subsequent surfactin biosynthesis. In this way, a promoter-independent molecular control mechanism could be used to control the availability of the surfactin-forming NRPS enzyme. The principle was applied in high cell-density bioreactor processes, which led to a decoupling of biomass formation and production phase. The physiological adaptations caused by the induction of the surfactin-forming NRPS and the associated surfactin production were studied in more detail using mass spectrometric proteome analyses.

## 2. Materials and Methods

### 2.1. Chemicals and Standards

Chemicals used in this study, if not otherwise stated, were purchased from Carl Roth GmbH & Co. KG (Karlsruhe, Germany). For quantitative measurements, surfactin standards (purity ≥ 98%) were provided by Sigma-Aldrich Laborchemikalien GmbH (Seelze, Germany). The ncAA O-methyl-L-tyrosine (purity ≥ 98%) was obtained from Thermo Scientific (Waltham, MA, USA). The glucose concentration in samples was quantified using an enzymatic assay kit (R-Biopharm AG, Darmstadt, Germany).

### 2.2. Cloning Procedure

Standard molecular methods were performed as previously described by Sambrook and Russel [35]. Oligonucleotides, plasmids and strains used for the cloning procedure are provided in Tables S1–S3. PCR reactions were carried out with PrimeStar Max DNA polymerase (TaKaRa Bio Europe SAS, Saint-Germain-en-Laye, France) by following the description of the manufacturer. Transformation of plasmid-based ligation mixtures was conducted with CaCl_2_ chemically competent *E. coli* strain TOP10. Extraction of plasmids were conducted with the NucleoSpin Plasmid EasyPure kit (Bioke, Leiden, The Netherlands) and they were sequenced by Macrogen Europe (Amsterdam, The Netherlands).

For the chromosomal integration of the amber stop codon, the *srfAA* integration site was cloned into the plasmid pJOE6743-1 via Gibson Assembly [36]. The oligonucleotides “srfAA ACT-4-TAG fwd” and “srfAA ACT-4-TAG rev” were used for codon replacement ACT::TAG within the *srfAA* integration site of the plasmid. Chromosomal integration of the orthogonal aaRS/tRNA mutant pair from *Methanococcus jannaschii* allowing the incorporation of OMeY into amber stop codons was possible according to the principle of LFH-PCR [14,32]. For this purpose, the backbone of the pBUA-P224 plasmid, including the aaRS/tRNA mutant pair and the upstream-located P_224_ promoter system, was fused with the flanking regions of the *amyE* integration locus using the backbone of the pKAM446 plasmid [15,32].

The principle of mannose-counter selection was applied for the construction of markerless *B. subtilis* mutant strains [37]. Therefore, the plasmid pJOE6743-1 with *srfAA* integration site and codon exchange ACT::TAG was transformed into the *B. subtilis* strain CT6, a derivative of *B. subtilis* BMV9 which lacks the *manPA* codon as previously described by Vahidinasab et al. [14] and exhibits a chromosomally integrated mannitol-inducible *comS-comK* cassette [38]. After chromosomal substitution (ACT::TAG) at codon position four, the aaRS/tRNA mutant pair was chromosomally integrated into the *amyE* gene locus. The following selection markers were added for mutant strain selection: 100 µg/mL ampicillin for *Escherichia coli*, and 100 µg/mL spectinomycin for *B. subtilis*.

### 2.3. Strain and Conditions for Cultivations

The *B. subtilis* strain BMV9 with inducible competence (P*_mtlA_*-*comS-comK*) was used for expanding the genetic code. The corresponding *B. subtilis* strain AH2 (Table S1) derived from this BMV9 strain was used for the subsequent studies.

For shake flask cultivation, pre-cultures were grown overnight in LB-medium (10 g/L tryptone, 5 g/L yeast extract and 5 g/L NaCl). The main cultures were inoculated at an initial optical density (OD_600_) of 0.1 in a chemically defined mineral salt medium containing 7.12 g/L Na_2_HPO_4_ × 2H_2_O, 4.08 g/L KH_2_PO_4_, 6.61 g/L (NH_4_)_2_SO_4_, 0.20 g/L MgSO_4_ × 7 H_2_O and 1 mL/L trace element solution (TES). TES contained 1.49 g/L Na_2_EDTA × 2H_2_O, 0.78 g/L CaCl_2_, 1.11 g/L FeSO_4_ × 7H_2_O and 0.17 g/L MnSO_4_ × H_2_O. The pH of the media used was adjusted to 7.0. In addition, 8 g/L of glucose was added as the sole carbon source. Cultivations were conducted in biological triplicates in baffled Erlenmeyer flasks at 37 °C and 120 rpm in an incubation shaker (Innova 44^®^R, Eppendorf AG, Hamburg, Germany).

### 2.4. Conditions for Fed-Batch Bioreactor Cultivations

The pre-cultures were prepared according to the method described by Klausmann et al. [27]. The first pre-culture was grown in LB medium, while the second pre-culture was cultured in a chemically defined mineral salt medium. The pre-cultures were grown in a New Brunswick™/Innova 44 incubator shaker (Eppendorf AG, Hamburg, Germany) at 37 °C with a shaking speed of 120 rpm.

The bioreactor experiments were performed in a 30-litre fermenter (ZETA GmbH, Lieboch, Austria) filled with 12 liters of batch medium containing 25 g/L glucose. The general conditions such as temperature, aeration, pH, pressure and pO_2_ were the same as described in Hiller et al. [22]. Modifications were made in terms of foam control. More specifically, a foam trap and antifoam agents were omitted, while a foam centrifuge was retained as the only protective mechanism against excessive foaming. The second pre-culture was used to inoculate the batch medium to an optical density (OD_600_) of 0.1. After inoculation, the cultivation process was performed until glucose was depleted, following with the initiation of feeding as described by Hiller et al. [22] using the pO_2_ monitoring established by Henkel et al. [39]. The feed used in this study was a 50% (*w*/*w*) glucose solution with a total volume of 6 L containing trace elements solution and magnesium sulfate [27]. The initial feeding rate F_0_ was calculated using Equation (1). This initial feeding rate was then applied in Equation (2) to achieve an exponential addition of the glucose solution at the feeding rate F at each time point t of the process.

(1) $$F_{0}=\left(\frac{\mu }{Y_{X/S}}+m\right)\times \left(\frac{C_{X,Batch}\;\times \;V_{0}}{C_{S,Feed}}\right)$$

(2) $$F(t)=F_{0}\;\times \;e^{\mu \;\times \;t}$$

In this context, F(t) is the feeding rate at every time point t of the exponential feeding process (kg/h), F_0_ is the initial feeding rate after the consumption of the batch glucose (kg/h), µ is the desired growth rate set to 0.25 1/h, Y_X/S_ the substrate to biomass conversion yield in the batch phase (g/g), m the maintenance term set to 0.05 g/(g × h), C_X,Batch_ the amount of biomass at feed start (g/L), V_0_ is the batch volume (L) and C_S,Feed_ the glucose concentration in the feed solution set to 500 g/L.

To induce surfactin production, the ncAA O-methyl-L-tyrosine (OMeY) was added at a rate of 0.25 1/h during the feeding process. Two different process variants were tested; in the first process variant, OMeY was added to the bioreactor to a final concentration of 0.75 mM once the culture reached an OD_600_ of 100. The feeding process was continued until a final volume of 6 L of feed solution was consumed, at which point the process was stopped.

In the second process variant, OMeY was added about four hours after the start of feeding. The aim was to achieve the same concentration of 0.75 mM in the bioreactor at a similar OD_600_ of 100 as in the first process. To compensate additionally dilution effects caused by the feeding process, OMeY was introduced into the glucose feed solution. The amount of the amino acid added was calculated to ensure a constant ratio of OMeY to OD throughout the process. Similar to the first variant, the process was stopped after 6 L of feed had been consumed.

### 2.5. Surfactin Quantification

Surfactin purification was performed as previously described by Geissler et al. [40]. In brief, a volume of 2 mL of cell-free supernatant was extracted three times with chloroform/methanol (2:1) before drying the pooled solvent layers in a rotary evaporator (10 mbar, 40 °C). After solubilization in 2 mL methanol, the sample was applied to a HPTLC silica plate in 6 mm bands. Chloroform/methanol/water (65:25:4) was used as mobile phase, and a migration distance of 60 mm was selected.

### 2.6. Calculation of Cell Dry Weight

Since a strain derived from *B. subtilis* BMV9 was used in this study, a correlation factor of 0.232 was used to convert optical density (OD_600_) to cell dry weight (CDW), as previously described by Hiller et al. [22].

### 2.7. Data Analysis

The calculations for the yields Y_P/S_ and Y_P/X_, which describe the conversion of glucose as substrate (S) and biomass (X) into surfactin as product (P) during the process time (t), the growth rate (µ) and the specific productivities q_P/S_ and q_P/X_, are described in the following equations. For the calculation of the performance parameters of surfactin production, the period of induction feeding with OMeY was used.

(3) $$Y_{P/X}=\frac{P}{X}|P={P(t}_{end})$$

(4) $$Y_{P/S}=\frac{P}{S}|P={P(t}_{end})$$

(5) $$Y_{X/S}=\frac{X}{S}|X={X(t}_{end})$$

(6) $$q_{P/X}=\frac{P_{max}}{X\times t}$$

(7) $$q_{P/S}=\frac{P_{max}}{S\times t}$$

(8) $$µ=\frac{ln{(CDW}_{1})-ln({CDW}_{2})}{\Delta t}$$

### 2.8. Protein Extraction, On-Bead Digestion and Peptide Purification for Proteome Analysis

A cell suspension of the *B. subtilis* strain AH2 was grown in mineral salt medium until the mid-exponential phase of about 2, before 25 mM of OMeY was added. The addition of water was used as a negative control. A volume of 5 mL was taken 10, 30 and 60 min after addition, before the cells were separated by centrifugation (7197× *g*, 5 min). *B. subtilis* cell pellets were lysed in lysis-buffer (4% SDS, 100 mM Tris HCl pH = 8.5). Lysates were cleared by centrifugation at 17,000× *g* for 15 min and protein concentrations of the supernatants were determined by the Bradford assay [41]. Supernatants were adjusted to a protein concentration of 1 µg/µg with 100 mM Tris HCl (pH 8.5). Proteins were extracted, cleaned, and digested with a modified SP3 (Single-Pot Solid-Phase-enhanced Sample Preparation) based sample preparation method [42]. Briefly, equal amounts of magnetic beads (SpeedBeads™ magnetic carboxylate modified particles; Cytiva, Marlborough, MA, USA; CAT No: 45152105050250 and 65152105050250) were activated by washing with 100 mM Tris HCl (pH = 8.5). Subsequently, activated magnetic beads were transferred into the lysates (25 µg total protein amount per sample) in a 1/10 (protein/magnetic beads, *w*/*w*) ratio. Samples were sonicated for 5 min (Elmasonic S 40, Elma Schmidbauer GmbH, Singen, Germany) to prevent aggregation of the magnetic beads. Proteins were bound to the magnetic beads by adding ethanol to a final concentration of 70% (*v*/*v*). Samples were incubated on a magnetic rack and magnetic beads were washed three times with 1 mL of 80% ethanol. Protein reduction and alkylation on the beads were performed in 0.2% SDC (sodium deoxycholate), 10 mM TCEP (tris(2-carboxyethyl)phosphine), 50 mM chloroacetamide and 50 mM Tris HCl (pH 8.5) in the dark (30 min, 56 °C, 1000 rpm). Proteins were digested with 50 ng LysC protease (FUJIFILM Wako Pure Chemical Corporation, Osaka, Japan) and 250 ng trypsin (Roche, Penzberg, Germany) at 36 °C and 700 rpm overnight. Supernatants were acidified to a final concentration of 2% with FA (formic acid). Magnetic beads and SDC-precipitates were pelleted by centrifugation (16,000× *g*). Peptides in the supernatant were concentrated and desalted on C18 stage tips [43]. Eluted peptides were dried under vacuum and dissolved in 0.2% FA and 2% acetonitrile for NanoLC-MS/MS analyses.

#### 2.8.1. NanoLC-MS/MS Analysis

NanoLC-ESI-MS/MS experiments were performed on an Ultimate 3000 nano-RSLC (Thermo Fisher Scientific, Waltham, MA, USA) coupled to an Exploris 480 mass spectrometer (Thermo Fisher Scientific, Waltham, MA, USA) using a Nanospray-Flex ion source (Thermo Fisher Scientific, Waltham, MA, USA). Peptides were concentrated and desalted on a trap column (5 mm × 30 µm, Thermo Fisher Scientific, Waltham, MA, USA) and separated on a 25 cm × 75 µm nanoEase MZ HSS T3 reversed phase column (100 Å pore size, 1.8 µm particle size; Waters, Milford, CT, USA) operated at constant temperature of 35 °C. Peptides were separated at a flow rate of 300 nL/min using a 65 min method with the following profile: 2–8% solvent B in 2 min, 8–30% solvent B in 32 min, 30–47% solvent B in 13 min, 47–96% solvent B in 3 min, isocratic at 96% solvent B for 5 min, 96–2% solvent B in 5 min and isocratic at 2% solvent B for 5 min. Solvents used were 0.1% formic acid (solvent A) and 0.1% formic acid in acetonitrile/H_2_O (80/20, *v*/*v*, solvent B). MS spectra (*m*/*z* = 300–1400) were detected in the Orbitrap at a resolution of 60,000 (*m*/*z* = 200) using a maximum injection time (MIT) of 50 ms and an automatic gain control (AGC) value of 3 × 106. Internal calibration of the Orbitrap analyzer was performed using lock-mass ions from ambient air as described in Olsen et al. [44]. MS/MS spectra of the top 30 peptide precursors per cycle were generated in the Orbitrap using high energy collision dissociation (HCD) fragmentation at a resolution of 15,000 and a normalized collision energy of 30. Further settings for MS/MS spectra included an isolation width of 1.6 Da, a MIT of 40 ms and an AGC value of 8 × 104.

#### 2.8.2. MS Data Analysis and Protein Quantification

Raw files were imported into MaxQuant [45] version 2.0.1.0 for protein identification and label-free quantification (LFQ) of proteins. Protein identification in MaxQuant was performed using the database search engine Andromeda [46]. MS spectra and MS/MS spectra were searched against *B. subtilis* (strain 168) protein sequence database downloaded from UniProt [47]. Reversed sequences as decoy database and common contaminant sequences were added automatically by MaxQuant. Mass tolerances of 4.5 ppm (parts per million) for MS spectra and 20 ppm for MS/MS spectra were used. Trypsin was specified as an enzyme and two missed cleavages were allowed. Carbamidomethylation of cysteines was set as a fixed modification and protein N-terminal acetylation and oxidation were allowed as variable modifications. The ‘match between runs’ feature of MaxQuant was enabled with a match time window of 0.7 min and an alignment time window of 20 min. Peptide false discovery rate (FDR) and protein FDR thresholds were set to 0.01. Statistical analysis was performed using Perseus version 1.6.14.0 [48]. Matches to contaminant (e.g., keratins, trypsin) and reverse databases identified by MaxQuant were excluded from further analysis. First, normalized LFQ values from MaxQuant were log2 transformed. Only proteins with a minimum of three valid LFQ quantifications in at least one group of replicate experiments (n = 3) in the data set were considered for the statistical analysis. Remaining missing values were imputed in R (https://www.r-project.org (accessed on 12 November 2024), version 3.6.2) using the QRILC (Quantile Regression Imputation of Left-Censored data) function in the imputeLCMD package (version 2.1) [49,50,51] with a tune sigma value of one. Significant changes in protein abundance were analyzed by a Welch’s *t*-test (two sided, S0 = 0.1) and corrected for multiple hypothesis testing using permutation-based FDR statistics (FDR = 0.05, 250 permutations).

## 3. Results

### 3.1. Development of a B. subtilis Production Strain for OMeY-Dependent Surfactin Production

For surfactin biosynthesis, which depends on the presence of the ncAA O-methyl-L-tyrosine (OMeY), a derivative of the surfactin-producing *B. subtilis* strain CT6, derived from BMV9—with chromosomally integrated mannitol-inducible *comK-comS* cassette—was used as the parental strain. Furthermore, transformation efficiency was not affected by effects on the expression of the native *comS* gene located in the *srfAB* gene locus.

In a first step, the third codon in the *srfAA* gene of the *srfA* operon, which encodes the surfactin-producing NRPS, was replaced by an amber stop codon according to the principle of mannose-counter selection. For a subsequent expansion of the genetic code, an aaRS/tRNA mutant pair derived from *Methanococcus jannaschii* and under control of the strong P_224_ promoter system was chromosomally integrated into the *amyE* gene locus, resulting in a selection marker-independent application of the genetic code expansion [32].

Subsequently, surfactin production in the engineered *B. subtilis* production strain, called AH2, was validated in shake flask cultivations with chemically defined mineral salt medium using different OMeY starting concentrations of 0, 0.25, 0.5, 0.75 and 1 mM.

**Figure 2:**
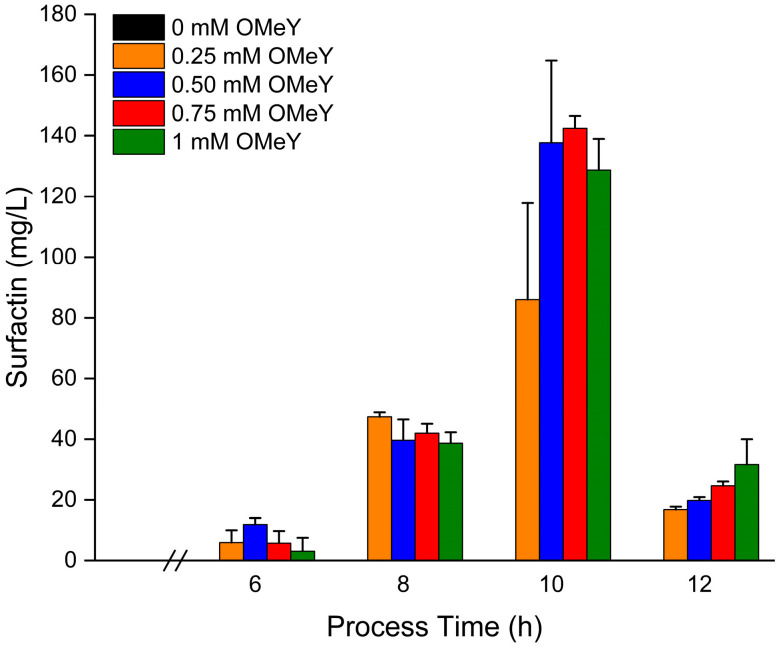
Validation of the correlation between surfactin production and the addition of OMeY. Shake flask cultures were performed using a mineral salt medium containing 8 g/L glucose and 0, 0.25, 0.5, 0.75 and 1 mM of OMeY. *B. subtilis* strain AH2 was cultured for 12 h. Samples were taken regularly for quantitative surfactin measurement. All cultivation approaches were performed in biological triplicates.

While no surfactin production could be detected during the entire cultivation process in the absence of OMeY, maximum surfactin concentrations of 136 ± 18 mg/L were achieved with 0.5 mM OMeY and higher OMeY concentrations after 10 h of cultivation (Figure 2). In contrast, surfactin levels of 86 ± 32 mg/L were measured at an initial concentration of 0.25 mM OMeY. Accordingly, a saturation of SrfAA protein translation based on the genetic code expansion was assumed when using approximately 0.5 mM of OMeY as an inductor. This observation was also be made with LB medium using a plasmid-based incorporation system [32]. However, after reaching a maximum level of surfactin after 10 h of cultivation, a decrease to approximately 23 ± 7 mg/L after 12 h was observed in all cultures.

Another aspect was the surfactin-mediated effect on cell growth. In this context, improved cell growth was observed in the non-producing *B. subtilis* cultures with 0 mM OMeY. In the exponential growth phase between 4 and 8 h of cultivation, cultures grown with 0.25 mM OMeY showed a 9% reduction in cell growth, and with 0.5 mM OMeY a 12% reduction, presumably as a result of higher surfactin-forming NRPS translation and subsequent surfactin biosynthesis. However, the surfactin-producing *B. subtilis* cultures in the presence of OMeY were able to achieve higher maximum biomasses than the non-producing cultures in absence of OMeY (0.25 mM—1.05 fold; 0.5 mM—1.08 fold; 0.75 mM—1.16 fold; 1 mM—1.17 fold).

### 3.2. Implementation of OMeY-Dependent Surfactin Production into Fed-Batch Bioreactor Cultivations

**Figure 3:**
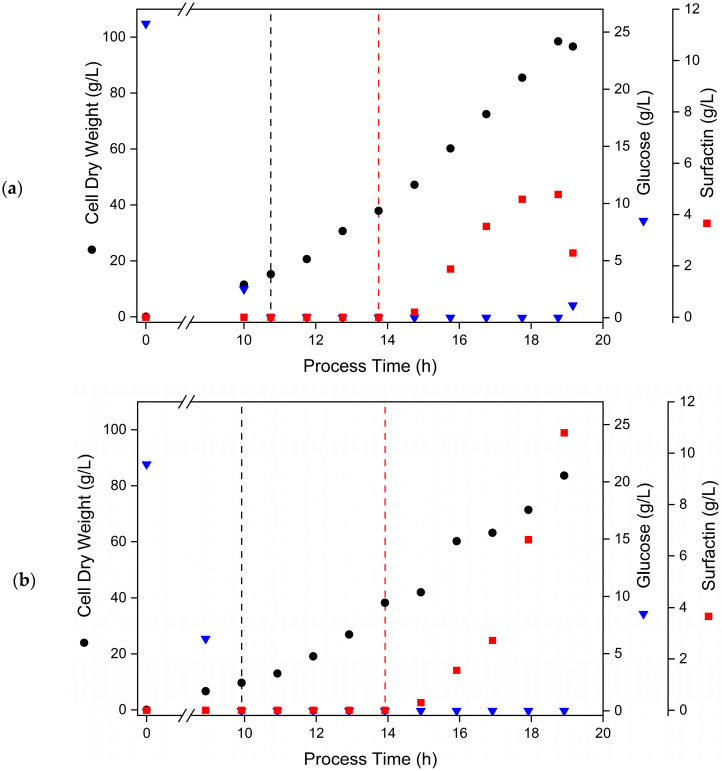
Development of bioreactor processes with feeding strategies allowing OMeY-dependent surfactin production. The engineered *B. subtilis* surfactin production strain AH2 was cultivated in a batch fermentation until the glucose was depleted and the feeding process was started (black dashed line). When the culture reached an OD_600_ of approximately 100, surfactin production was activated by adding OMeY as an inducer (red dashed line) at a final concentration of 0.75 mM (**a**) followed by volume-associated co-feeding (**b**). The entire bioreactor process was stopped when the 6-litre glucose feed solution was consumed.

After confirming OMeY-mediated stimulation of surfactin production in shake flask cultivations, *B. subtilis* strain AH2 was used for fed-batch bioreactor cultivation processes. Since the constructed strain was derived from the sporulation-deficient *B. subtilis* 3NA and BMV9, respectively, a high cell-density bioprocess was performed as previously described by Klausmann et al. [27] and Hiller et al. [22] (Figure 3).

In the first approach, the AH2 strain was cultivated in a batch phase up to a CDW of 15.2 g/L (10.75 h of cultivation) before the glucose was depleted and a feeding procedure with 0.25 1/h was initiated. After reaching a biomass concentration CDW of 37.9 g/L, surfactin production was induced by adding OMeY at a final concentration of 0.75 mM. Subsequently, an accumulation of surfactin was observed, whereas no surfactin was measured prior to OMeY addition. In this way, a maximum surfactin concentration of 4.8 g/L was reached after 5 h of induction. Thereafter, a reduction in biomass formation was observed, while further feeding procedure led to a dilution effect of biomass and surfactin with an associated glucose accumulation. Due to the feeding-derived dilution of surfactin, a final titer to 2.5 g/L was measured at the end of the bioprocess (Figure 3a).

To further improve the ncAA-based surfactin bioproduction process, a feeding-associated OMeY addition was performed. In this way, dilution effects caused by the feeding process were compensated. Accordingly, OMeY was premixed into the glucose feed solution to ensure a constant ratio of ncAA and biomass throughout the process. More specifically, the first feeding phase was started after 10 h of cultivation, achieving a CDW of 9.7 g/L. When the feeding phase lasted for 4 h and a CDW of 38.3 g/L was reached, an initial addition of OMeY was performed, allowing a final concentration of 0.75 mM, before an in-feed OMeY addition was installed. In this way, a constant volume-associated OMeY concentration could be achieved. As a consequence, an increase in surfactin titer was observed during the entire feeding-associated surfactin activation phase. Finally, a maximum surfactin titer of 10.8 g/L was reached after 5 h of induction. Remarkably, no reduction in the surfactin increase could be observed, suggesting that extended cultivation periods also result in higher maximum surfactin titers (Figure 3b).

Table 1 provides an overview of the production parameters achieved in the fed-batch bioreactor processes described.

### 3.3. Effects of OMeY-Associated Surfactin Induction on B. subtilis Physiology

**Figure 4:**
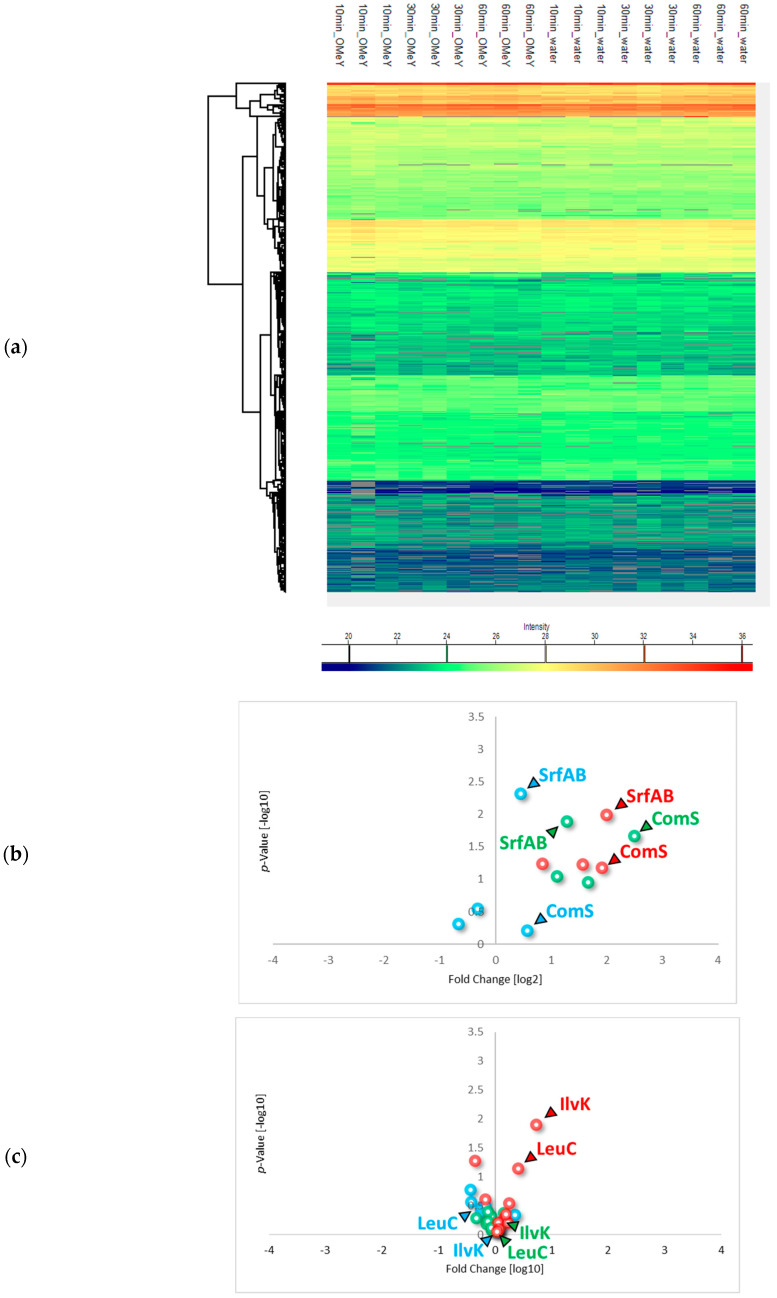

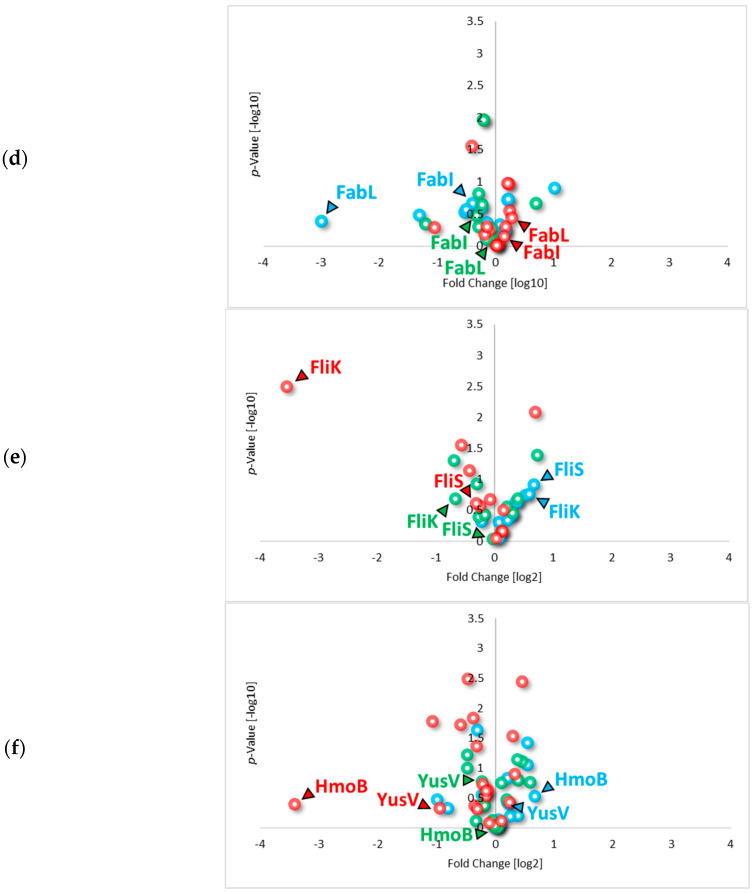
Proteome analysis regarding physiological adaptations based on OMeY-derived induction of surfactin production. (**a**) Heatmap without normalization as an overview of the protein signal intensities determined between the OMeY-induced and control time points. Volcano plots represent different groups of proteins, namely SrfA proteins for surfactin biosynthesis (**b**), enzymes for biosynthesis of branched-chain amino acids (**c**) and fatty acids (**d**), proteins associated with motility (**e**) and iron acquisition (**f**), and their changes in abundance after 10 (blue), 30 (green) and 60 min (red) of inducting surfactin production.

In order to characterize the influence of the induction of surfactin production by the addition of OMeY, which is not exclusively incorporated into the amber stop codon of the engineered *srfAA* gene locus, a proteome analysis was performed. For this purpose, *B. subtilis* strain AH2 was cultivated in shake flasks to a mid-exponential growth phase with an OD_600_ of 2 before OMeY was added at a final concentration of 0.75 mM. The addition of the same volume of water served as a negative control. Samples were then taken 10, 30 and 60 min after addition (Figure 4a).

Changes in the abundance of proteins in the *B. subtilis* surfactin production strain AH2 were monitored by mass spectrometric analysis. In this context, an accumulation of the SrfA proteins, including the competence anti-adaptor protein ComS encoded in the *srfAB* gene locus, was detected (Figure 4b). Accordingly, polar effects appear to be present in the expression of the *srfA* operon.

In addition, the biosynthesis of surfactin requires an intracellular supply of precursor molecules, including amino acids and fatty acids. Accordingly, enzymes associated with the biosynthesis of branched-chain amino acids were found to be increased in the abundance after OMeY-based surfactin induction (e.g., branched-chain amino acid aminotransferase IlvK and 3-isopropylmalate dehydratase LeuC). This observation is reasonable as the surfactin structure contains four leucine and one valine molecule, indicating a higher requirement for branched-chain amino acids as precursor molecules (Figure 4c). In addition, a slight increase in proteins associated with fatty acid biosynthesis (e.g., enoyl-acyl carrier protein reductases FabI and FabL) was observed (Figure 4d). In this way, cellular adaptations in terms of a higher availability of biosynthetic enzymes for both precursor groups, namely for the peptide moiety and fatty acid chain, could be found.

Beyond this, more global adaptive processes associated with the OMeY-mediated stimulation of surfactin-forming NRPS and subsequent surfactin biosynthesis were identified. More specifically, changes were detected in motility-associated proteins, particularly flagellar proteins such as the flagellar hook-length control protein FliK and the chaperone for flagellin export FliS (Figure 4e). Thus, while after 10 min of OMeY addition most of the flagellar proteins showed slightly increased abundance compared to the negative control, a decrease in abundance was observed over time compared to non-activated surfactin-producing cell cultures, indicating an adaptation of *B. subtilis* motility to a more sessile state in presence of surfactin.

As surfactin also possesses chelating properties and enables the removal of metals from contaminated soil and sediments [52], the surfactin-producing *B. subtilis* cells show a slight reduction in the abundance of proteins associated with iron acquisition (e.g., heme monooxygenase HmoB and the ABC transporter for siderophores YusV) (Figure 4f). Consequently we can assume that the chelating properties of surfactin have a positive effect on the acquisition of metal ions, so that *B. subtilis* cells are able to reduce the provision of additional uptake mechanisms.

## 4. Discussion

As a proven expression host for a variety of proteins, *B. subtilis* has been used in several previous studies for the controlled expression of target genes. A common strategy is the transcriptional fusion of the target gene with either an inducible or constitutively active promoter system. However, since native upstream regions can also have a regulatory influence on the biosynthesis of the target protein, further strategies to control the protein biosynthesis need to be explored. For example, substitution of the native quorum sensing-regulated *srfA* promoter region for the constitutively active P*_veg_* promoter region did not lead to increased surfactin bioproduction in all *B. subtilis* production strains tested [30]. In this context, genetic code expansion enables native initiation of target gene expression including the transcription process. Therefore, orthogonal protein translation systems need to be integrated into the expression host. The use of an orthogonal aaRS/tRNA mutant pair enables codon-specific incorporation of ncAA, such as targeted O-methyl-L-tyrosine (OMeY) [31]. In this study, a translation system using an aaRS/tRNA mutant pair from *Methanococcus jannaschii*, previously shown by Tian et al. [32], was used. To reduce the impact of reprogramming non-targeted codons in the host, the amber stop codon (UAG), as the least frequently used codon in bacteria, is commonly used for genetic code expansion approaches [53]. In *B. subtilis* strain 3NA and BMV9, the parental strains of the engineered *B. subtilis* strain AH2 used in this study, 587 amber stop codons are present in 4276 coding DNA sequences [54].

This reprogramming leads to competition between the native release factor and the orthogonal protein translation system. Accordingly, strategies to improve the efficiency of ncAA incorporation could include reduced expression of the native release factor, high expression or engineering of the ncAA incorporation machinery and screening for alternative orthogonal aaRS/tRNA mutant pairs [32,55,56]. In addition, Xu et al. [53] systematically optimized a *B. subtilis* strain suitable for genetic code expansion by exchanging 22 UAG stop codons of essential genes, resulting in a reduction of codon competition and further optimization of *B. subtilis* as a microbial cell factory.

However, in contrast to inducible promoter expression systems with external inducers, such as the inducible promoter systems P*_xylA_* (xylose), P*_mtlA_* (mannitol) and P*_grac_* (IPTG), the principle of genetic code expansion shows a very strict and dose-dependent protein biosynthesis by addition of external ncAA [33,57]. This principle makes it possible to control enzyme biosynthesis by adding the ncAA as an inducer and thus indirectly fine-tune the underlying promoter strength externally. As a result, the number of target proteins per cell can be specifically adjusted by adding ncAA.

Using this approach, absolutely no surfactin could be determined in shake flask cultivation with *B. subtilis* production strain AH2 in the absence of OMeY as an inducer, whereas surfactin production could be detected by OMeY addition (Figure 2). However, since the aaRS/tRNA mutant pair was chromosomally integrated for reasons of genetic stability, a lower incorporation of OMeY compared to a plasmid-based system is reasonable, as previously shown by Tian et al. [32]. Accordingly, only a maximum surfactin titer of 136 ± 18 mg/L could be measured, while the surfactin-producing domesticated *B. subtilis* strain JABs24 and the *B. subtilis* wild-type strain DSM10T produced 568.8 and 112.4 mg/L, respectively, in the same chemically defined mineral salt medium and 8 g/L glucose as carbon source [19]. Nevertheless, a genetically robust system might be more suitable for upscaled bioproduction processes. In this context, conventional bioreactor processes for surfactin production are prone for excessive foam formation, resulting in loss of culture media and cells associated with over-foaming [58]. While in situ product removal approaches have been developed, such as the installing of a foam fractionation column or a foam trap [26,59], the use of foam centrifuges and antifoam agents to remove foam has been described in several bioprocesses [22,27].

However, the application of antifoam agents is associated with a reduction in oxygen transport, resulting in impaired oxygen supply to the cell culture [60], while continuous foam formation reduces the availability of nutrients and cellular productivity within the foam fraction [59]. Accordingly, approaches have been developed to decouple biomass formation and surfactin production. For this purpose, in various studies, the native *srfA* promoter was exchanged for inducible expression systems such as the synthetic IPTG-inducible P_spac_ promoter system [61] and the native oxygen-sensitive P*_nasD_* promoter [15]. However, the limitations of these studies include either uncontrolled activation of strong promoter systems or basal expression levels under non-activating conditions. In contrast, this study provided an application of the principle of genetic code expansion, allowing an inducible surfactin production phase that was uncoupled from biomass formation initiated by the addition of OMeY (Figure 3a). Thus, a single OMeY addition during the feeding procedure of a fed-batch bioreactor cultivation with the *B. subtilis* production strain AH2 resulted in a maximum surfactin concentration of 4.8 g/L after 5 h post-induced cultivation. However, as the titer of surfactin produced after OMeY induction was significantly lower compared to conventional high cell-density cultivations with a feeding rate of 0.25 1/h and a comparable *B. subtilis* production strain (BMV9) reaching up to 36 g/L [22], a modified fed-batch procedure was developed. Therefore, in addition to a single pulse to induce surfactin production, a subsequent feeding-associated OMeY addition was introduced. As a result, higher inducer availability was present during the surfactin production phase, resulting in a maximum titer of 10.8 g/L, corresponding to a 2.25-fold improvement (Figure 3b). In general, a production time of 5 h was used, although an increase in surfactin concentration was still observed at the end of the process time, indicating higher maximum titers with extended production phase. In this way, the OMeY-driven surfactin production process could compete with conventional surfactin production processes. However, when comparing de novo surfactin production under the same conditions (feeding rate of 0.25 1/h, starting OD_600_ of 100 and about 5 h of cultivation), the parental *B. subtilis* strain BMV9 produced about 20 g/L surfactin [22]. Accordingly, further improvements in the genetic code expansion are required. For this purpose, a higher incorporation rate of ncAA is needed, which addresses the general competition between the peptide chain release factor RF1 and the ncAA-incorporating aminoacyl-tRNA. According to Tian et al. [32], the expression system derived from the pBUA-P224 plasmid in *B. subtilis* achieved only 58.5% of the native gene expression. However, as the orthogonal aaRS/tRNA system was chromosomally integrated with the aim of increasing genetic stability, the number of incorporation machinery available was reduced, resulting in a decrease in ncAA incorporation efficiency. Nevertheless, the study demonstrated saturation of ncAA-based surfactin production with 0.5 to 0.75 mM OMeY in shake flask cultures, which also could be found by Tian et al. in complex LB medium cultures [32]. To further increase the efficiency of OMeY incorporation, a higher copy number of the orthogonal aaRS/tRNA system or a lower availability of the release factor might be useful.

Another limitation in the upscaled high cell-density fed-batch bioreactor fermentations shown in this study is the high amount of inducer substance required for efficient induction of enzyme expression, which makes the application of ncAA-based genetic code expansion less attractive for further scaling approaches. To solve this problem, a two-step induction mechanism could be attractive, such as an ncAA-inducible T7 polymerase-based expression system.

Regarding physiological effects observed after the induction of surfactin production by stimulating SrfAA protein translation, several physiological adaptation processes were identified. In particular, an increase in SrfA protein levels, including the anti-adapter protein for competence development, ComS, was detected (Figure 4b). Accordingly, not only the targeted SrfAA protein, but also the translation of the entire *srfA* operon was affected by the incorporation of the ncAA OMeY. It can therefore be assumed that polar effects are present in the expression process of the surfactin-forming NRPS.

Due to the biosynthesis of surfactin, precursor molecules are required. A corresponding demand could be determined indirectly through increased abundances of the proteins involved in precursor metabolism. Specifically, Leu- and Ilv-proteins, which address the pathways for branched-chain amino acids, and Fab-proteins, which are required for fatty acid biosynthesis, were found in increased abundances after the induction of surfactin production (Figure 4c,d).

In addition, a physiological adaptation was observed that may be more related to the structural properties of surfactin. For example, flagellar proteins associated with *B. subtilis* motility were found to be reduced in abundance after surfactin induction (Figure 4e). Since surfactin supports swarming in *B. subtilis* by reducing the surface tension of the surrounding fluid [62,63], the accumulation of surfactin molecules could support general microbial motility. As a result, reduced flagella formation could occur as a compensation to save metabolic energy.

In addition, a reduction in the abundances for proteins associated with iron acquisition was observed (Figure 4f). In this context, the chelating properties of surfactin have previously been described to enable the binding of metal ions [64,65]. Accordingly, extracellular surfactin could support the uptake of iron and other metal ions required for vital physiology of *B. subtilis*. If this is the case, the balance between native siderophores and the uptake system need to be restored to ensure a controlled intracellular homeostasis of metal iron in the *B. subtilis* production strain.

## 5. Conclusions

A first step towards the development of methods for the precise molecular regulation of NRPS molecules per cell was taken in this work. It was shown that non-canonical amino acids can be used for expression control in high cell-density fermentations. The engineered *B. subtilis* surfactin production strain AH2 showed a higher cell growth rate and a tight gene control without measurable expression prior to induction. However, the productivity achieved was still relatively low compared to conventional fermentation methods. Further investigation of optimal growth rates and induction conditions could improve the performance of this system. Further strain engineering, including higher efficiency of ncAA incorporation, may help to address crucial bottlenecks regarding an externally controllable cell by genetic code expansion. In this context, the combination of different induction systems could improve ncAA-based surfactin production, while the expression of other orthogonal aaRS/tRNA mutant pairs could extend the levels of control by combining cell growth and bioproduction in an exemplary setting.

## Figures and Tables

**Table 1:** Summary of parameter from OMeY-mediated surfactin production.

**Process Parameters**	**Fed-Batch Process with Single OMeY Addition**	**Fed-Batch Process with Feed-Associated OMeY Addition**
Maximum Surfactin [g/L]	4.8	10.8
Y_P/S_ [g/g]	0.018	0.086
Y_P/X_ [g/g]	0.026	0.13
Y_X/S_ [g/g]	0.52	0.45
q_P/S_ [g/(g × h)]	0.0034	0.017
q_P/X_ [g/(g × h)]	0.0048	0.026

## Data Availability

The original contributions presented in this study are included in the article/Supplementary Materials. Further inquiries can be directed to the corresponding author.

## Supplementary Materials

The following supporting information can be downloaded at: https://www.mdpi.com/article/10.3390/microorganisms13020353/s1, Table S1: Strains used in this study; Table S2: Plasmids used in this study; Table S3: Oligonucleotides used in this study. Excel file S1: Mass Spectrometric Proteome Analysis after OMeY-based Surfactin Induction.
