# Supplementary material for: Genetic Code Expansion for Controlled Surfactin Production in a High Cell-Density Bacillus subtilis Strain
Source: Microorganisms. 2025 Feb 6;13(2):353. doi: 10.3390/microorganisms13020353 (PMC11858380; doi:10.3390/microorganisms13020353)
Supplement: Supplementary file 1 [file microorganisms-13-00353-s001.zip › 05-02-2025_Revised Supplementary Material.pdf]

Genetic code expansion for controlled surfactin production in high cell-density strain *Bacillus subtilis* BMV9

Alexander Hermann[1,†], Eric Hiller[1,†], Philipp Hubel[2], Lennart Biermann[1], Elvio Henrique Benatto Perino[1], Oscar Paul Kuipers[3], Rudolf Hausmann[1], Lars Lilge[1,*]

1 Department of Bioprocess Engineering, Institute of Food Science and Biotechnology, University of Hohenheim, Stuttgart, Germany; alexander.hermann@uni-hohenheim.de; eric.hiller@uni-hohenheim.de; lennart.biermann@uni-hohenheim.de; eperino@uni-hohenheim.de; rudolf.hausmann@uni-hohenheim.de; lars.lilge@uni-hohenheim.de.
2 Core Facility Hohenheim, Mass Spectrometry Core Facility, University of Hohenheim, Stuttgart, Germany; philipp.hubel@uni-hohenheim.de
3 Department of Molecular Genetics, University of Groningen, AG Groningen, The Netherlands; o.p.kuipers@rug.nl
† These authors contributed equally to this work.
* Correspondence: lars.lilge@uni-hohenheim.de; Tel.: +49 71145924736

Table S1. Strains used in this study.

Strain	Genotype	Reference
B. subtilis BMV9	*spo0A3*; *sfp+*; *trp+*; Δ*manPA*	[14]
B. subtilis CT6	Derived from *B. subtilis* BMV9; *hisI*::P$_{mtlA}$-*comK-comS*	This study
B. subtilis AH1	Derived from *B. subtilis* CT6; *srfAA* [ACT::TAG (10-12 nt)]	This study
B. subtilis AH2	Derived from *B. subtilis* AH1; *amyE*::P224-aaRS/tRNA-*spcR*	This study

Table S2. Plasmids used in this study.

Plasmid	Genotypic features	Reference
pJOE6743-1	ori$_{pUC18}$, *bla*, *spc*, *manP*, *ter-lacI-lacZα-ter*	[38]
pJOE6743-1-srfAA-4-TAG	ori$_{pUC18}$, *bla*, *spc*, *manP*, +1000 bp upstream region-*srfAA* (1589 bp) with ACT::TAG (10-12 bp)	This study
pBUA-P224	pHT01-OMeY derivate with mutant tRNA under the control of P$_{224}$ promoter	[33]
pKAM446	Integration vector for genomic integration in the *amyE* locus: *ori*$_{pUC18}$, *bla*, *rop*, *ermC*, *amyE'*-[*ter*-P$_{srfAA}$-*lacZ*, *spcR*]- '*amyE*	[15]

Table S3. Oligonucleotides used in this study.

Oligonucleotide	Sequence (5´ → 3´)	Comments
srfAA up fwd link pJOE6743-1 Gibson	ATTCTAATGAATTGCCATTCAAAAAGTAAGTAAGTGATG ATACGTATC	Gibson Assembly of *srfAA* integration site into the initial mannose-counter-selection plasmid pJOE6743-1
srfAA down rev link pJOE6743-1 Gibson	TTCTGTGGATAACCGTATTACCCATGATAGCTGTAATCAT CC	
pJOE6743-1 screening rev	GATTTTTGTGATGCTCGTCAG	Linearization of the plasmid pJOE6743-1
pJOE6743-1 backbone fwd	TAATACGGTTATCCACAGAATCAG	
pJOE6743-1 backbone rev	GAATGGCAATTCATTAGAATGAATATTTC	Confirmation of Gibson Assembly
srfAA ACT 4 TAG fwd	GACAATATGGAAATATAGTTTTACCCTTTAACGG	Integration of codon substitution against amber stop codon
srfAA ACT 4 TAG rev	CCGTTAAAGGGTAAAACTATATTTCCATATTGTC	
srfAA substitution seq fwd	CTTTTTAAGTGTAGTACTTTGGGC	Sequencing of correct codon substitution
amyE up fwd	GTTCATCATTATCTTATATTACTGCATCAG	Amplification of the amyE integration locus
amyE rev	GCTTAAGCCCGAGTCATTATATAAAC	
pBUA fwd	ctaaatttttatctaaagtgaatttaggaggc	Amplification of the backbone of plasmid pBUA-P_{224}
pBUA rev	gacgcgtgacgtgaaaaaag	
amyE up rev link pBUA	gcctcctaaattcactttagataaaaatttagCGATCAGACCAGTTTT TAATTTGTG	LFH-PCR construction for integration of aaRS/tRNA pair into the *amyE* locus
amyE fwd link pBUA	gcttttttcacgtcacgcgtcTAATAAGAATTCCTGCAGCCCT	
